# Systemic Administration of Insulin Receptor Antagonist Results in Endothelial and Perivascular Adipose Tissue Dysfunction in Mice

**DOI:** 10.3390/cells10061448

**Published:** 2021-06-09

**Authors:** Bartosz Proniewski, Anna Bar, Anna Kieronska-Rudek, Joanna Suraj-Prażmowska, Elżbieta Buczek, Krzysztof Czamara, Zuzanna Majka, Izabela Czyzynska-Cichon, Grzegorz Kwiatkowski, Karolina Matyjaszczyk-Gwarda, Stefan Chlopicki

**Affiliations:** 1 Jagiellonian Centre for Experimental Therapeutics (JCET), Jagiellonian University, Bobrzynskiego 14, 30-348 Krakow, Poland; bartosz.proniewski@jcet.eu (B.P.); anna.bar@jcet.eu (A.B.); anna.kieronska@jcet.eu (A.K.-R.); joanna.suraj@jcet.eu (J.S.-P.); elzbieta.buczek@jcet.eu (E.B.); krzysztof.czamara@jcet.eu (K.C.); zuzanna.majka@student.uj.edu.pl (Z.M.); iza.czyzynska@jcet.eu (I.C.-C.); grzegorz.kwiatkowski@jcet.eu (G.K.); karolina.matyjaszczyk@jcet.eu (K.M.-G.); 2 Faculty of Pharmacology, Jagiellonian University Medical College, Grzegorzecka 16, 31-531 Krakow, Poland; 3 Faculty of Chemistry, Jagiellonian University, Gronostajowa 2, 30-387 Krakow, Poland

**Keywords:** endothelial function, magnetic resonance imaging, perivascular adipose tissue, insulin receptor antagonist

## Abstract

Hyperglycemia linked to diabetes results in endothelial dysfunction. In the present work, we comprehensively characterized effects of short-term hyperglycemia induced by administration of an insulin receptor antagonist, the S961 peptide, on endothelium and perivascular adipose tissue (PVAT) in mice. Endothelial function of the thoracic and abdominal aorta in 12-week-old male C57Bl/6Jrj mice treated for two weeks with S961 infusion via osmotic pumps was assessed in vivo using magnetic resonance imaging and ex vivo by detection of nitric oxide (NO) production using electron paramagnetic resonance spectroscopy. Additional methods were used to analyze PVAT, aortic segments and endothelial-specific plasma biomarkers. Systemic disruption of insulin signaling resulted in severe impairment of NO-dependent endothelial function and a loss of vasoprotective function of PVAT affecting the thoracic as well as abdominal parts of the aorta, however a fall in adiponectin expression and decreased uncoupling protein 1-positive area were more pronounced in the thoracic aorta. Results suggest that dysfunctional PVAT contributes to vascular pathology induced by altered insulin signaling in diabetes, in the absence of fat overload and obesity.

## 1. Introduction

Vascular endothelium represents a single cellular layer and is recognized as an autocrine/paracrine/endocrine multifunctional organ that plays a crucial role in maintaining body homeostasis, while endothelial dysfunction has not only pathophysiological and diagnostic but also therapeutic significance [1,2]. Indeed, the endothelium is involved in the regulation of vessel permeability, blood transport, vascular tone, new blood vessel formation [3,4,5] and plays a critical role in pro-inflammatory, pro-thrombotic, and angiocrine signaling. Dysfunction of the endothelium is involved in most, if not all, human diseases, either as a primary determinant of pathophysiology or as a victim of collateral damage that can amplify the pathophysiology of a disease [1,6]. In particular, type 2 diabetes (T2D) is currently one of the most frequently reported causes of endothelial and vascular dysfunction resulting from increased hyperglycemia [7] and disruption of insulin signaling [8], ultimately leading to the development of cardiovascular diseases [9].

In animal models, elevated glucose was first suggested to be a common pathway leading to endothelial dysfunction in insulin-dependent diabetes mellitus and hyperglycemia-induced insulin resistance in the 1990s [10]. However, the role of perivascular adipose tissue (PVAT) in early diabetes is less recognized, despite the fact that it plays a critical role in the pathogenesis of atherosclerosis, hypertension, aortic aneurysm, where PVAT increases in volume and becomes dysfunctional, losing its thermogenic capacity and secretes pro-inflammatory cytokines with altered cellular composition and molecular characteristics [11,12]. PVAT positively regulates vascular function through the production of factors such as adiponectin, and its vasoprotective role is attenuated in obesity [13]. Interestingly, the role of PVAT has been connected with vascular pathologies, mostly in the context of obesity, fat overload and insulin resistance [14], but not with early hyperglycemia or specific alterations in insulin signaling. One possible reason for this limitation in understanding the role of PVAT in early hyperglycemia is the fact that diabetes research has been historically conducted with both pharmacological and genetic preclinical models that do not fully recapitulate the human condition, including pharmacologically induced T2D with streptozocin (STZ), or genetic model of type 2 diabetes of db/db mice. Recently, a novel experimental approach to mimic the pathophysiology of T2D model was proposed, based on the administration of the insulin receptor antagonist (IRA), the S961 peptide, which has been shown to induce transient hyperglycemia and hyperinsulinemia in mice [15] and rats [16], as well as to elicit NADPH-dependent endothelial nitric oxide synthase (eNOS) uncoupling in rabbits [17] and microvascular complications in mice [18]. Given the accumulating evidence suggesting that PVAT’s structural and functional alterations might contribute to endothelial dysfunction [19] and that brown adipose tissue (BAT) is able to rescue the endothelium [14], here we aimed to comprehensively verify the effects of a short-term, 2-week treatment with the S961 peptide, not only on endothelial but also PVAT function in the TA and AA in mice, using the state-of-the-art method for in vivo assessment of endothelial function, based on magnetic resonance imaging (MRI) with subsequent ex vivo analysis of the endothelium and PVAT.

## 2. Materials and Methods

### 2.1. Animals

Studies were performed in 12-week-old male C57BL/6Jrj mice (Janvier Labs), fed a control diet (AIN93G, ZooLab). Age was selected so that the endothelium was unaffected by biological ageing and the study was performed on male mice to be comparable to previously published data on the vascular effects of S961 treatment in mice [18]. Mice were kept in collective cages with free access to food and water in a controlled environment in accordance with the Guide for the Care and Use of Laboratory Animals of the National Academy of Sciences (NIH publication No. 85–23, revised 1996), as well as the Guidelines for Animal Care and Treatment of the European Community. All experiments were approved by the Ethics Local Committee of Jagiellonian University (permit number 283/2019, Krakow, Poland). IRA (S961 peptide, a gift from Novo Nordisk), with the sequence GSLDESFYDWFERQLGGGSGG- SSLEEEWAQIQCEVWGRGCPSY and a disulfide bridge connecting the two cysteine residues [20], was dissolved in 0.9% sodium chloride with 10% DMSO and infused for 14 days at a dose of 0.57 mg/kg/day via osmotic mini-pumps (model 1002, Alzet, Cupertino, CA, USA) implanted subcutaneously under general anesthesia (S961 group, n = 11). The dose used was established based on preliminary experiments and previously published studies [18]. Control animals (VEH group, n = 8) were implanted with osmotic pumps filled with a vehicle, to take account for any negative effects pump implantation might have on endothelial function. Mice were randomly assigned to each group and sample analysis were performed blinded. The size of a given experimental group in particular assessments are reported in the legends of the corresponding graphs.

### 2.2. Blood Sampling and Tissue Collection

One week after osmotic pumps were implanted, a drop of blood from the tail was taken from each animal to measure glucose levels using a standard glucometer and monitor the effectiveness of S961 administration. After two weeks of infusion, MRI was performed and the following day mice were anesthetized (100 mg/kg ketamine + 10 mg/kg xylazine, i.p.) and whole blood glucose measurement from the tail were repeated. Next, 50 µL of blood without anticoagulant was taken from the right ventricle of the heart, centrifuged at 2500× *g*, at a temperature of 4 °C for 15 min to collect serum, used for C-peptide 2 assessment with an ELISA kit according to manufacturer’s manual (Rat/Mouse C-Peptide 2 ELISA, Merck Millipore, Darmstadt, Germany), while the remainder of the blood was drawn and collected in tubes containing 10% solution of dipotassium ethylenediaminetetraacetic acid (K_2_EDTA, Aqua-Med, Lodz, Poland: 1 μL of EDTA/100 μL of blood). Whole blood was used to measure morphology (Abc Vet, Horiba Medical, Montpellier, France) and glycated haemoglobin (Hb1Ac, ABX Pentra, Horiba Medical, Kyoto, Japan). A subset of whole blood was mixed with MS-SAFE Protease and Phosphatase Inhibitor Cocktail (Sigma–Aldrich, Poznan, Poland) in a ratio of 100:1 and centrifuged at 664× *g*, at a temperature of 4 °C for 10 min to isolate plasma for biomarker analysis. The remainder of whole blood was centrifuged at 1000× *g*, at a temperature of 4 °C for 5 min. Plasma samples were deep frozen at −80 °C for high performance liquid chromatography (HPLC) measurements of nitrate (NO_3_^−^) and nitrite (NO_2_^−^) concentrations by ENO-20 NOx Analyzer (Eicom, Kyoto, Japan) and measurements of metabolic and biochemical parameters using the Horiba system. The remaining red blood cells (RBCs) were deep frozen at −80 °C for measurements of reduced and oxidized glutathione (GSH and GSSG, respectively) concentrations by capillary electrophoresis as described by Hempe et al. [21] and nitrosylhemoglobin content in RBCs using electron paramagnetic resonance (EPR), as described by Kramkowski et al. [22]. Aortas with PVAT were collected for further ex vivo assessments as described below. Moreover, interscapular adipose tissue and epididymal adipose tissue were collected for Raman spectroscopy analysis, described below.

### 2.3. Assessment of Endothelium-Dependent Vasodilation by MRI In Vivo

MRI experiments were performed using a 9.4T scanner (BioSpec 94/20 USR, Bruker Biospin, Ettlingen, Germany) 2 weeks after osmotic pumps were implanted. During the experiment, mice were anaesthetized using isoflurane (Aerrane, Baxter Sp. z o. o., Warsaw, Poland: 1.5 vol%) in an oxygen and air (1:2) mixture, and imaged in the supine position. Heart function (rhythm and electrocardiogram), respiration and body temperature (maintained at 37 °C using circulating warm water) were monitored using a Monitoring and Gating System (Small Animal Instruments Inc., San Diego, CA, USA).

Endothelial function in vivo was assessed by 2 techniques described previously [23,24,25], as endothelium-dependent response to acetylcholine (Ach) administration and flow-mediated dilatation (FMD) in response to reactive hyperaemia, considered a gold standard in studies on endothelial dysfunction in humans. Additionally, endothelium-independent response to i.v. sodium nitroprusside (SNP) administration was measured. Response to injection of Ach (Sigma-Aldrich, Poznan, Poland: 50 μL, 16.6 mg/kg, i.p.; the dose of Ach was based on the previous study [23]) and SNP (Sigma-Aldrich, Poznan, Poland: 1 mg/kg, i.v.), was analyzed in the TA and AA, whereas FMD after short-term (5 min) occlusion in the femoral artery (FA) induced by a home-made vessel occluder, described elsewhere [24].

Vasomotor responses were examined by comparing two, time-resolved 3D images of the vessels prior to and 30 min after intraperitoneal Ach administration or after five minutes’ vessel occlusion. The optimal time to measure Ach-induced vasorelaxation and the optimal time for vascular occlusion to measure FMD response were chosen based on our previous work [23] and of other authors [26]. Three-dimensional images of the aorta were positioned on the sagittal view of the mice, whereas 3D images of the FA were positioned on the coronal view of the mice, on the right hind limb. Images were acquired using the cine IntraGate™ FLASH 3D sequence and reconstructed with the IntraGate 1.2.b.2 macro (Bruker Biospin, Ettlingen, Germany). The analysis was performed using ImageJ software 1.46r (NIH, Bethesda) and scripts written in Matlab (Mathworks Inc., Natick, MA, USA), in the smaller hyperstack of the AA (10 slices in diastole, from renal arteries down), the TA (10 slices in diastole, from celiac artery up) and the FA (7 slices). All cross-sectional areas of vessels at each slice were obtained using thresholding segmentation and exported to Matlab, where vessel volumes were reconstructed and calculated.

### 2.4. Assessment of NO Production in the Aorta Using EPR

For measurements of eNOS-dependent NO production, EPR spin-trapping with diethyldithiocarbamic acid sodium salt (DETC, Sigma-Aldrich, Poznan, Poland) was used ex vivo, as described previously [27,28], with minor modifications. Isolated aorta was divided into two parts, namely abdominal and thoracic sections. The TA and AA were cleared from surrounding tissue and the respective PVAT samples saved for Raman and EPR analysis, described below. Parts of the aorta were opened longitudinally and preincubated in Krebs-HEPES buffer for 30 min at 37 °C. Next, DETC (3.6 mg) and FeSO_4_·7H_2_O (2.25 mg) were separately dissolved under argon gas bubbling in two 10 ml volumes of ice-cold Krebs–HEPES buffer and were kept under gas flow on ice until used. After pre-incubation, a spin trap (125 µL of FeSO_4_·7H_2_O and 125 µL of DETC—final concentration of the colloid: 285 µM) and calcium ionophore A23187 (Sigma-Aldrich, Poznan, Poland: the final concentration 1 µM) were added to the parts of the aorta. Subsequently, incubation for 90 min at 37 °C was started. Finally, dried aorta was weighed and frozen in liquid nitrogen (suspended in fresh buffer) into the middle of a 400 μL column of Krebs–Hepes buffer and stored at −80 °C until measured. EPR spectra were obtained using an X-band EPR spectrometer (EMX Plus, Bruker, Rheinstetten, Germany). Signals were quantified by measuring the total amplitude of the NO-Fe(DETC)_2_ and are expressed in arbitrary units/mg of tissue and normalized to the respective controls.

### 2.5. Assessment of Biomarkers of Endothelial Dysfunction in Plasma by microLC/MS-MRM

Assessment of protein biomarkers of endothelial dysfunction was performed using the microLC/MS-MRM method as described and used by our group previously [14,25,29,30,31]. The panel included biomarkers of various aspects of endothelial dysfunction such as: glycocalyx disruption: syndecan-1 (SDC-1) and endocan (ESM-1); endothelial inflammation: soluble vascular cell adhesion molecule 1 (sVCAM-1), soluble intercellular adhesion molecule 1 (sICAM-1), soluble form of E-selectin (sE-sel), soluble form of P-selectin (sP-sel); endothelial permeability: angiopoietin 1/2 (Angpt-1/2), soluble form of fms-like tyrosine kinase (sFLT-1) and hemostasis: von Willebrand factor (vWF), tissue plasminogen activator (t-PA) and plasminogen activator inhibitor 1 (PAI-1). The panel included also adiponectin (ADN), adrenomedullin (ADM), soluble Tie-2 (sTie-2), annexin A5 (ANXA5), thrombospondin-1 (THBS-1), thrombin-activatable fibrinolysis inhibitor (TAFI).

A UPLC Nexera system (Shimadzu, Kyoto, Japan) connected with a highly sensitive mass spectrometer QTrap 5500 (Sciex, Framingham, MA, USA) was used. During sample preparation, the studied mouse material was subjected to proteolytic digestion using porcine trypsin to achieve unique and reproducible peptide sequences, applied as the surrogates of the proteins suitable for LC-MS/MS analyses.

### 2.6. Immunohistochemical Characteristics of PVAT and Endothelium of the TA and AA

Formalin-fixed and paraffin-embedded thoracic and abdominal parts of the aorta with PVAT were cut into 5-μm slices. Antigen retrieval was performed according to the standard protocol. The area of the brown and white adipose tissue in PVAT was assessed by double staining using perilipin-1 and uncoupling protein 1 (UCP1) antibodies. To visualize the PVAT area, anti–perilipin-1 antibody was used (ab61682, Abcam, Cambridge, UK), whereas the area of BAT was identified by UCP1 staining (ab209483, Abcam, Cambridge, UK). Next, sections were incubated with the secondary antibodies FITC-conjugated donkey anti-goat IgG (705-454-003, Jackson ImmunoResearch, West Grove, PA, USA) and Cy3-conjugated donkey anti-rabbit (711-165-152, Jackson ImmunoResearch, West Grove, PA, USA). To define the total area of PVAT, perilipin-1 and UCP1 immuno-positive areas were summed.

To characterize the functional differences between brown and white adipose tissue, lectin I, adiponectin, endothelial NO synthase, and phosphorylated endothelial NO synthase (Phospho-eNOS) were stained. To visualize glycocalyx of microvessels in PVAT, biotinylated Lectin I (B-1105-2, Vector Laboratories, Burlingame, CA, USA) was used. The inflammatory processes in endothelium were characterized as ratio of eNOS (610296, BD Biosciences, San Diego, CA, USA) and phospho-eNOS—Ser1177 antibody (9571, Cell Signaling Technology, Denver, CO, USA). Then, sections were incubated with the secondary biotinylated-conjugated goat anti-mouse and goat anti-rabbit IgG antibody, respectively (111-065-003, Jackson ImmunoResearch, West Grove, PA, USA). To assess the function of PVAT, adiponectin (Ab 62551, Abcam, Cambridge, UK) followed a secondary biotinylated-conjugated goat anti-rabbit IgG antibody. Then, the slices were incubated with VECTASTAIN Elite ABC-HRP Kit (PK-6100, Vector Laboratories, Burlingame, CA, USA) and diaminobenzidine (Sigma-Aldrich, Poznan, Poland) to obtain the color reaction. Subsequently, the cross-sections of the TAs and AAs with PVAT were photographed (×100 magnification) and images were acquired using an AxioCam MRc5 digital camera and an AxioObserver 22 D1 inverted fluorescent microscope (Carl Zeiss Jena, Oberkochen, Germany) or BX51 microscope (Olympus, Tokyo, Japan). Before analysis in the immuno-stained pictures with Lectin I and Phospho-eNOS non-adipose tissue fragments (aorta wall, muscles, lymph nodes) were manually excised. Image segmentation was performed automatically using Ilastik (developed by the Ilastik team, with partial financial support of the Heidelberg Collaboratory for Image Processing, HHMI Janelia Farm Research Campus and CellNetworks Excellence Cluster), where the algorithm classifies pixels based on identical criteria of image properties (color, edge and texture) assigned to different categories (e.g., UCP1-immunopositive), defined on representative images by an histology expert, allowing for unbiased quantitative analysis of large dataset. The pixels corresponding to the UCP1(Cy3 channel), as well as perilipin-1 (FITC channel) were quantitatively determined using ImageJ software 1.46r. The mean number of pixels representing UCP1 and perilipin-1 were counted using ImageJ software. The results were expressed as the ratio of brown adipose tissue area (number of UCP1 immunopositive pixels/PVAT area. Moreover, Lectin I and adiponectin was shown as the number of immunopositive pixels/PVAT area. When the activated endothelium was demonstrated as a ratio of eNOS immunopositive area to Phospho-eNOS immunopositive pixels normalized to PVAT area.

### 2.7. Oxidant Properties of PVAT by EPR Spectroscopy

Fragments of the thoracic and abdominal PVAT were used for assessment of reactive oxygen species production using EPR detection of a cell permeable cyclic hydroxylamine spin probe, 1-hydroxy-3-methoxycarbonyl-2,2,5,5-tetramethylpyrrolidine (CMH, Enzo Life Sciences, Farmingdale, NY, USA) oxidation, that has been used for detection of intracellular O_2_^•−^ in cultured cells and tissue samples in cardiovascular studies [32], as described in [33]. Briefly, samples were homogenized in Tris-EDTA buffer containing 0.25 M sucrose with a 1:6 lung/buffer (mg/μL) ratio using Dounce tissue grinder with a glass or pestle. 50 μL of the homogenate was added to 450 μL of KHB containing 100 μM DTPA. In a 1.5 mL tube (in a total volume of 100 μL), to 98 μL of homogenate in KHB, 2 μL of CMH of 10 mM stock were added to obtain a final concentration of 0.2 mM. Samples were incubated for 20 min at 37 °C in a water bath and placed on ice to stop the reaction. Next, these were loaded in a capillary tube and EPR spectra were recorded for 360 s at room temperature. The amount of oxidized CMH spin probe (CM^•^) was quantified using the slope linear regression line, normalized to the protein concentration of each sample. Some samples were run in duplicate to test the contribution of superoxide radical (via a 20 min preincubation with superoxide dismutase, 500 U/mL) and a matched blank sample with CMH KHB containing sucrose buffer was used to take into account spin probe auto-oxidation.

### 2.8. Lipid Characteristic of Adipose Tissue by Raman Spectroscopy

Brown adipose tissue was resected from the interscapular area (iBAT) and white adipose tissue was collected from the adipose tissue attached to the epididymis and testicle (eWAT). Periaortic PVAT samples of TA and AA were extracted from the descending aorta after the aortic arch and from the part of the artery lying in the abdominal cavity, respectively. Samples were prepared as described previously [34]. In brief, adipose tissue sections were washed with NaCl isotonic solution to remove blood traces and placed directly onto CaF_2_ slides. The Raman spectra of all samples were excited with a 532 nm laser and collected with a 20× air objective (S Plan Flour, Nikon Instruments Inc., Melville, NY, USA, NA = 0.45) of a confocal WITec Alpha300 Raman spectrometer (WITec, Ulm, Germany). The single spectra of 32 accumulations and the integration time of 0.5 s were acquired using the maximum laser power (∼28 mW). For each studied adipose tissue type, at least 5 single Raman spectra were collected and then were normalized in the 1800–400 cm^−1^ spectral range and averaged over all mice. All Raman spectra underwent routine data preprocessing including baseline-correction using autopolynomial of degree 3 and an automatic cosmic ray removal procedure. Data preprocessing was performed using the WITec Project Plus software. The averaging and presentation of Raman spectra were performed using the OriginPro 9.1 (OriginLab Corporation, Northampton, MA, USA) program. For calculations of the integral intensity of the bands at ca. 1660 and 1445 cm^−1^ enabling estimation of the lipid unsaturation degree (I_1660_/I_1445_) and the band at ca. 1750 cm^−1^ enabling estimation of the triacylglycerol level (I_1750_) the OPUS 7.2 (Bruker Optics Ltd., Ettlingen, Germany) program was used. The results were tested by analysis of variance performed using the Prism software to characterize quantitatively the differences in unsaturation of lipids in various types of the adipose tissue.

### 2.9. Statistical Analysis

Data are presented as mean ± SEM or mean ± SD unless otherwise stated, and plotted using GraphPad Prism 8.2.1 software (GraphPad Software Inc., La Jolla, CA, USA). All quantitative results were statistically analyzed applying the adequate parametric tests or non-parametric calculations. Results were considered statistically significant at *p* values equal to or below 0.05. Detail description can be found under the corresponding graphs and tables.

## 3. Results

### 3.1. Effects of Short-Term Administration of IRA in Mice—Basic Characteristics

The body weight in the S961-treated group decreased significantly (28.2 vs. 27.4 g, *p* < 0.05, 2-way ANOVA (time and type of treatment being the factors), repeated measures with Dunnett’s test) after 2-week administration of IRA, accompanied by the significant reduction in white adipose tissue around the mesenteric vascular bed. There were no significant differences in the spleen/body weight ratio or heart rate between S961-treated mice as compared with the vehicle-treated mice (data not shown).

Administration of S961 (0.57 mg/kg/day) resulted in an increase in blood glucose levels that was already detectable after one week of treatment (29.2(1.2) vs. 8.4(0.3) mmol/L in vehicle-treated mice, *p* < 0.0001) and remained constant until the end of the 2-week treatment (Table 1). At the 2-week endpoint of the study this was associated with a profound increase in HbA1c and various effects on biochemical and metabolic markers in the blood (Table 1). Importantly, S961-treated mice presented with an increased level of triglycerides (TG) and mildly decreased fraction of high-density lipoproteins (HDL) cholesterol. Slight elevation of alanine aminotransferase (ALT), but without changes in aspartate aminotransferase (AST) and a decrease in plasma creatinine were also noted. As regards blood count parameters, administration of S961 resulted in significant changes of red blood cell-related indices (RBC count, hemoglobin concentration, hematocrit, mean corpuscular volume, mean corpuscular hemoglobin concentration) without other changes (Table 2).

### 3.2. Effects of Short-Term Administration of IRA in Mice on Vascular Function and NO Production

**Figure 1:**
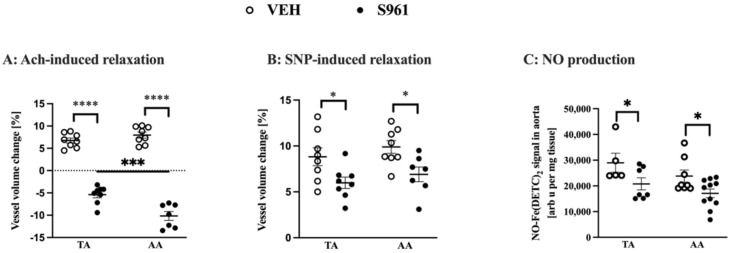
Effects of S961 treatment on vascular function of the thoracic and abdominal aorta. (**A**): Assessment of endothelium-dependent vascular response to acetylcholine (Ach) 30 min after administration in the thoracic aorta (TA, VEH n = 8, S961 n = 7) and abdominal aorta (AA, VEH n = 8, S961 n = 7) (**B**): Assessment of endothelium-independent vasodilation induced by sodium nitroprusside (SNP) 30 min after iv administration in thoracic aorta (TA, VEH n = 8, S961 n = 8) and abdominal aorta (AA, VEH n = 8, S961 n = 7). (**C**): Nitric oxide (NO) production in isolated TA (VEH n = 5, S961 n = 7) and AA (VEH n = 8, S961 n = 11), without PVAT. (**A**,**B**): significance assessed by Two-way ANOVA (aorta part and treatment being the two factors), normality confirmed by Shapiro-Wilk test, variance by F-test, with post hoc Sidak test. (**C**): significance assessed by two-sided t-test with Welch’s correction (normality was assessed using the Shapiro–Wilk test). * *p* < 0.05, *** *p* < 0.001, **** *p* < 0.0001.

After 2-weeks of infusing C57BL/6Jrj mice with S961 peptide, Ach-induced vasodilation in vivo in the TA as well as in the AA (Figure 1A) was completely lost. Simultaneously, the Ach response changed to paradoxical vasoconstriction (volume changes of the TA: −5.39% in comparison to 6.76% in vehicle-treated mice; and AA: −10.16% in comparison to 7.97% in vehicle-treated mice). The endothelium-independent vasodilation induced by SNP measured in vivo in the TA and AA was also slightly attenuated (Figure 1B). Additionally, in S961-treated mice, vasodilatation after the FA occlusion was also significantly impaired (6.9% vs. 29.5% in the control group, unpaired *t*-test *p* < 0.001, n = 4 (S961) and n = 3 (VEH)). Nitric oxide production in the isolated aorta (*p* < 0.005), was significantly decreased in S961-treated mice in the TA and in the AA confirming impaired NO-dependent function in the thoracic as well in abdominal aortic segments in S961—treated as compared with vehicle-treated groups (Figure 1C).

### 3.3. Effects of Short-Term Administration of IRA in Mice on Systemic NO Bioavailability and Protein Biomarkers of Endothelial Dysfunction

**Figure 2:**
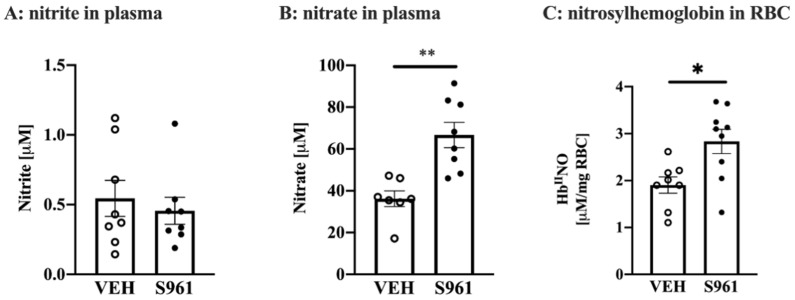
Effects of S961 treatment on plasma concentration of nitrite, nitrate and nitrosylhemoglobin content. Plasma concentration of (**A**): nitrite (VEH n = 8, S961 n = 8), (**B**): nitrate (VEH n = 7, S961 n = 8), (**C**): nitrosylhemoglobin (Hb^II^NO) content in red blood cells (RBC) (VEH n = 8, S961 n = 9). Statistical significance tested using two-sided Student’s t-test with Welch’s correction, normality confirmed by Shapiro–Wilk test, variance by F-test. Results shown as mean +/− SE. * *p* < 0.05, ** *p* < 0.01.

Infusion of S961 IRA in C57Bl/6Jrj mice for 2 weeks did not affect NO_2_^−^ (Figure 2A), but increased NO_3_^−^ plasma concentration (Figure 2B) and nitrosylhemoglobin content in RBCs (Figure 2C).

**Figure 3:**
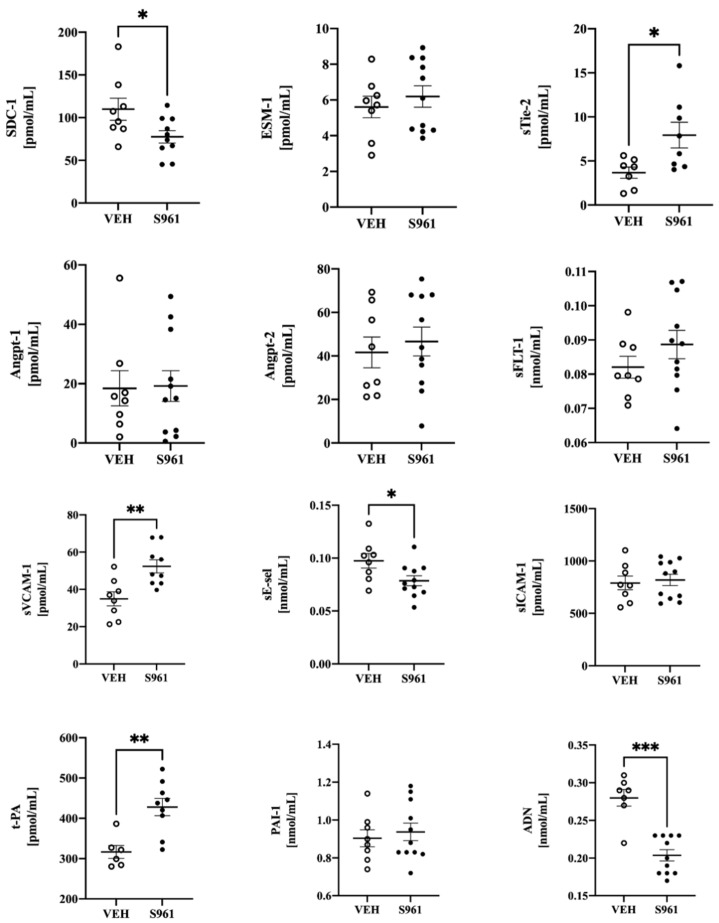
Effects of S961 treatment on selected plasma biomarkers of endothelial dysfunction. Biomarkers of glycocalyx disruption: syndecan-1 (SDC-1), endocan (ESM-1); endothelial permeability: soluble Tie-2 (sTie-2), angiopoietin 1 and 2 (Angpt-1/2), soluble fms-like tyrosine kinase (sFLT-1); endothelial inflammation: soluble form of E-selectin (sE-sel), soluble intercellular adhesion molecule 1 (sICAM-1), soluble vascular cell adhesion molecule 1 (sVCAM-1); hemostasis and others: tissue plasminogen activator (t-PA), plasminogen activator inhibitor 1 (PAI-1) and adiponectin (ADN). VEH n = 6–8, S961 n = 8–11. Statistical significance tested using two-sided Student’s *t*-test with Welch’s correction, normality confirmed by Shapiro–Wilk test, variance by F-test. Results shown as mean +/− SE. * *p* < 0.05, ** *p* < 0.01, *** *p* < 0.005.

Simultaneously, oxidant stress in S961-treated mice was confirmed in RBCs as evidence by reduced ratio of GSH/GSSG (see Table 1). In mice treated with S961 for 2 weeks, only few among biomarkers of endothelial dysfunction (Figure 3) displayed significant changes in plasma concentration, including the biomarker of glycocalyx disruption (SDC-1, but not ESM-1), the biomarker of endothelial permeability (sTie-2) with no apparent effect on other biomarkers of endothelial permeability (Angpt-1, Angpt-2, sFLT-1). There were signs of mild endothelial inflammation (sVCAM-1, sE-sel) and activation of t-PA without changes in PAI-1, and a fall in ADN in S961-infused mice as compared with mice given vehicle treatment. Other measured biomarkers did not show significant differences.

### 3.4. Effects of Short-Term Administration of IRA in Mice on PVAT

**Figure 4:**
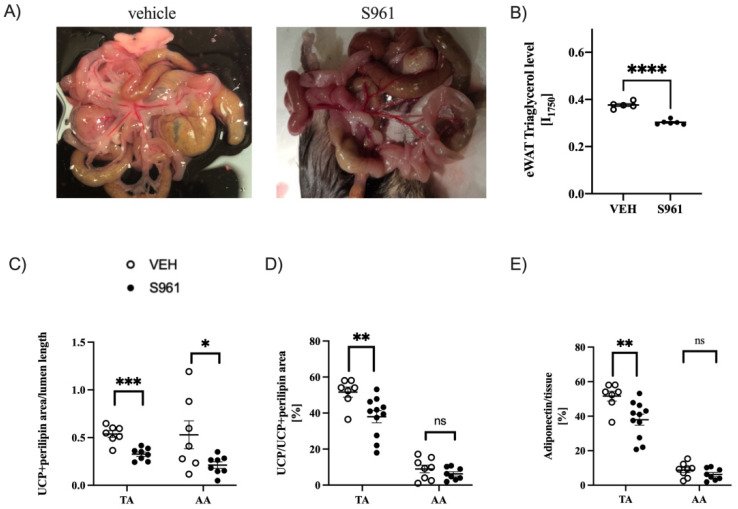
Effects of S961 treatment on fat depots amount and perivascular adipose tissue (PVAT). (**A**) images of the mesenteric arteries from vehicle (VEH)- and S961-treated mice, (**B**) Raman spectroscopy analysis of the intensity of the 1750 nm^−1^ band, representative of triacylglycerol content in epididymal white adipose (eWAT) tissue (VEH n = 5, S961 n = 6), (**C**) analysis of PVAT amounts in the thoracic (TA, VEH n = 7, S961 n = 8) and abdominal (AA, VEH n = 7, S961 n = 8) region, expressed as the total area uncoupling protein-1 (UCP1) and perilipin-1 positive staining normalized to vessel lumen, (**D**) immunohistochemical analysis of the ratio of UCP1 expression to UCP1 + perilipin-1 expression in PVAT of TA (VEH n = 7, S961 n = 11) and AA (VEH n = 8, S961 n = 8), representing the ratio of PVAT showing BAT-like characteristics, (**E**) decreased adiponectin expression in the TA (VEH n = 7, S961 n = 11) but not in AA (VEH n = 8, S961 n = 8). Statistical significance tested using two-sided Student’s t-test with Welch’s correction, normality confirmed by Shapiro–Wilk test, variance by F-test. not statistically significant (ns), * *p* < 0.05, ** *p* < 0.005, *** *p* < 0.001, **** *p* < 0.0001.

Similarly to the mesenteric vessels depicted on photographs (Figure 4A), administration of the S961 peptide to mice for a 2-week period resulted in marked decrease in PVAT content. This unexpected observation was in line with increased circulating TGs and with the observation of significant decrease in triacylglycerol levels in the eWAT tissue (Figure 4B). There was a decreased amount of PVAT in both brown and white fat deposits as evidenced by perilipin-1 and UCP-1 positive staining (Figure 4C). There was an initial difference in the PVAT of TA and AA in the area of UCP1-expression, due to the underlying differences in the morphology of these fat deposits. Interestingly, S961-treated mice had significantly decreased expression of UCP1 protein expression in the PVAT of TA section only (Figure 4D). Similarly, the significantly decreased of adiponectin expression was observed only in the PVAT of TA (Figure 4E).

**Figure 5:**
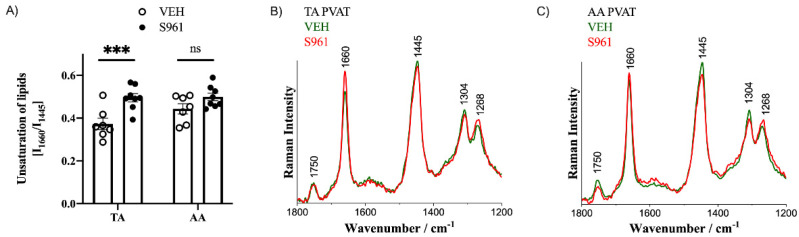
Effects of S961 treatment on adipose tissue chemical characteristics by Raman spectroscopy. Analysis (**A**) and averaged Raman spectra (**B**,**C**) of the lipid unsaturation degree (I_1660_/I_1444_) in TA and AA PVAT (in C57Bl/6 mice given vehicle (green; n = 5) or S961 (red; n = 5) via osmotic pumps for 2 weeks. Statistical significance tested using two-sided Student’s *t*-test with Welch’s correction, normality confirmed by Shapiro–Wilk test, variance by F-test: *** *p* < 0.005.

Moreover, PVAT surrounding the TA, and to a lesser extent the AA, exhibits increased content of unsaturated lipids (Figure 5), manifested as an increased intensity of Raman bands at 1660 and 1268 cm^−1^ assigned to the C=C stretching and =C–H deformation vibrations, respectively, and a decreasing intensity of the band at 1445 cm^−1^, due to the CH bending vibrations [36] and, thus, the integral intensity ratio of bands at 1660/1445 cm^−1^ was chosen to assess the lipid unsaturation.

**Figure 6:**
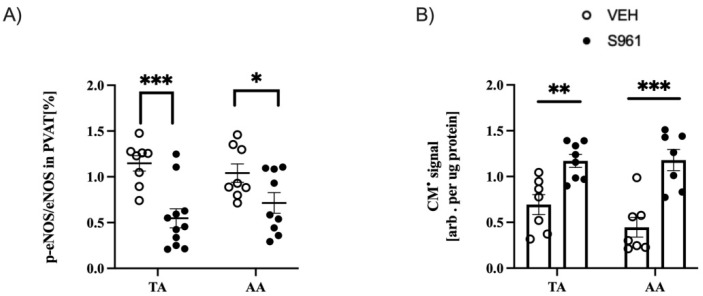
Effects of S961 treatment on eNOS activity and ROS production in PVAT and adiponectin expression. (**A**) Reduction eNOS activity in both TA (VEH n = 8, S961 n = 11) and AA (VEH n = 8, S961 n = 9) parts and (**B**) increased reactive oxygen species activity in PVAT expressed as spin probe oxidation in both TA (VEH n = 7, S961 n = 8) and AA (VEH n = 7, S961 n = 7). Statistical significance tested using two-sided Student’s t-test with Welch’s correction, normality confirmed by Shapiro–Wilk test, variance by F-test: not statistically significant (ns), * *p* < 0.05, ** *p* < 0.005, *** *p* < 0.001.

In contrast to a differential response in the TA vs. AA as regards, UCP1 protein, adiponectin and lipid unsaturation, immunohistochemical analysis in the TA and AA and the surrounding PVAT have shown similar degree of dysfunctional NO/ROS balance of PVAT, manifested by a modified redox balance. Activity of eNOS (ratio of p-eNOS/eNOS) in both PVAT depots was significantly lower in S961-treated mice (Figure 6A). Reactive oxygen species production was increased in the S961-treated mice (*p* < 0.0001) in both PVAT depots to similar degree, as evidenced by time-dependent oxidation of CMH and quantification of the CM^•^ radical formation (Figure 6B).

## 4. Discussion

Previously, S961 was shown to induce endothelial dysfunction and eNOS uncoupling in conduit arteries in rabbits after 7 days of treatment [17]. In contrast, S961 failed to induce endothelial dysfunction in the resistance vasculature at the end of a 4-week treatment regimen, based on in vivo measurements of bradykinin-induced fall in blood pressure even though there was higher superoxide production, 3-nitrotyrosine expression, lower phosphorylated eNOS expression [18]. The authors concluded that, despite the fact that S961-infusion model in mice resembled many of the clinical manifestations of T2D, this model did not result in endothelial dysfunction in mice. Here, we demonstrated, to our knowledge for the first time, that short-term administration of IRA (S961 peptide at 0.57 mg/kg/day for 2 weeks) in C57Bl/6Jrj mice, resulted in robust endothelial dysfunction in the conduit arteries, as well as PVAT dysfunction, the latter not analyzed in S961-treated animals previously that short-term 2-week-long infusion of IRA (S961, given at a higher dose of 0.57 mg/kg/day as in [18]) resulted in the development of profound endothelial dysfunction in young C57Bl/6Jrj male mice as evidenced by the substantially impaired acetylcholine- and flow-mediated vasodilation in the TA and AA, and in the FA, respectively. Endothelial dysfunction in S961-treated mice was also confirmed by a decreased nitric oxide production in the TA and AA, measured ex vivo using EPR spectroscopy [27]. Endothelial dysfunction in S961-trated mice was associated with shrinkage of brown type of PVAT, a fall in adiponectin expression in PVAT surrounding the thoracic aorta, as well as shrinkage of white type PVAT consistent with systemic lipolysis. Finally, S961-treated mice presented homogenously decreased eNOS activity and increased ROS production in PVAT surrounding the thoracic and abdominal aorta. Importantly, in vivo measurements were performed in the presence of intact PVAT surrounding the studied vessel, suggesting an impact of the PVAT secretory function not only on endothelial-dependent but also on endothelium-independent function of the vasculature in vivo, further supporting the unique value of in vivo measurements of endothelial function using MRI-based methodology [23,24,25] to demonstrate altered vascular reactivity in the S961-treated mice. Surprisingly, in vivo endothelium-independent vasodilatation in the S961-treated mice was compromised. These findings are in line with human in vivo studies suggesting that smooth muscle cells are also dysfunctional in T2D [36,37].

Recently, we have demonstrated that short-term (2 weeks) feeding of mice with a HFD resulted in insulin resistance (as evidenced by glucose tolerance test (GTT)) and in severe endothelial dysfunction in the abdominal, but not in the thoracic aorta. The difference in endothelial function along the aorta in response to HFD was ascribed to the distinct PVAT composition, with brown adipose tissue-like characteristics in the TA and white adipose tissue-like characteristics in the AA, as characterized by histology and immunohistochemistry and Raman spectroscopy [14]. The unique aspect about that particular study, was that it showed for the first time that even such a “short-term” HFD feeding induces endothelial dysfunction, contrary to previously published data describing endothelial dysfunction in long-term (>8 weeks) treatment.

In the present work, we analyzed the effects of a similar short-term treatment (2-week administration of IRA) and demonstrated profound effects on endothelial function in the aorta. However, in contrast to the HFD model, acetylcholine-induced vasodilation was impaired to a similar extent in both TA and AA, and PVAT surrounding both the TA and AA become dysfunctional in S961-treated mice. Accordingly, in contrast to the HFD, where endothelial function in the TA was resistant to early HFD-induced dysfunction, in S961-treated mice it was not the case, pointing out to profound effects of altered insulin signaling in both types of PVAT depots, even in the absence of fat overload and obesity after as short-term treatment as 2 weeks with S961. Comprehensive characterization of PVAT in the TA and AA, including decreased phosphorylation of eNOS (s1177) in PVAT previously shown also as a feature of PVAT alterations in HFD mice [38], clearly showed that S961 administration induced dysfunction and oxidative stress in PVAT, irrespective to the localization in TA or AA. Importantly, altered NOS/ROS balance featuring PVAT dysfunction in the TA and AA might contribute to vascular dysfunction induced by insulin receptor antagonism, possibly through the attenuated anti-contractile effects of PVAT [39].

On the other hand, S961 induced robust hyperglycemia and severe increase in HbA1c to the levels similar as in advanced-aged diabetic db/db mice [40,41], robust increase in GTT (much more pronounced than in the HFD model) and alterations in various RBC-related indices including increased GSH/GSSH ratio in RBCs. Interestingly, the connection between diabetic erythropathy and endothelial dysfunction was suggested recently [42], and could be relevant in the context of the profound endothelial dysfunction in the S961-treated animals. In contrast, other systemic effects of IRA administration (increased ALT, decreased plasma levels of creatinine, that latter ascribed to increased urination) seem unlikely to contribute to vascular dysfunction in S961-treated mice.

In previous studies, an increased nitrosative stress (expression of 3-nitrotyrosine), superoxide production by NADPH-oxidase activity and eNOS uncoupling in the endothelium of the aorta, was suggested to contribute to vascular dysfunction of conduit arteries in S961-treated rabbits [18]. Of note, changes in eNOS expression in the S961-treated mice could also at least in part originate directly from insulin receptor blockade, as in mice with conditional deletion of the insulin receptor gene limited to endothelial cells, eNOS expression in the aorta was decreased by 62% [43]. Similar finding was described in tamoxifen-induced insulin receptor knockout mice [44].

In our previous work, we reported that PVAT in the AA and TA displayed a different profile of lipid unsaturation, showing striking similarities between PVAT in the TA and iBAT, as well as PVAT in the AA and eWAT [14]. Accordingly, PVAT in the AA and TA shows a predominantly white and brown phenotype, respectively [45,46]. In the present work, insulin receptor inhibition by S961 caused extensive dysfunction of PVAT function featured by alteration in NOS/ROS balance in the TA and AA that was however associated with a differential transformation in the thoracic and abdominal PVAT depots. As such, the AA PVAT exhibited mainly lipolysis, whereas the TA PVAT, without insulin required for sustained physiological function of adipose tissue, underwent a functional reorganization, losing the brown adipose tissue functionality (UCP1) and losing its vasoprotective function (adiponectin) that might be functionally linked with pro-inflammatory response usually linked to increased lipid unsaturation. These results stay in line with a previous report showing that insulin receptor knock-out or inhibition, results in unrestrained lipolysis of WAT, leading to increased non-saturated fatty acids in the circulation [47]. Moreover, Sakaguchi et al. [44] described that the insulin receptor is critical in adipocyte maintenance, as tamoxifen-induced knockout of the insulin receptor leads to rapid loss of white and brown fat due to increased lipolysis and adipocyte apoptosis.

Taken together, our results uncovered that PVAT homeostasis is dependent on insulin signaling, a phenomenon that has been not appreciated previously. In particular, the restricted capacity for adaptive thermogenesis of brown-like PVAT in TA seems to represent an important feature of vascular dysfunction induced by insulin receptor blockade. Accordingly, robust hyperglycemia-induced endothelial dysfunction and altered insulin signaling-induced PVAT dysfunction seem both responsible for severe vascular dysfunction occurring just after 2 weeks of administration of S961 in mice.

In the present work vascular dysfunction induced by S961 was associated with increased nitrate and nitrosylhemoglobin, related possibly due to the nitrosative stress reported previously in this model [18]. However, regarding changes in plasma concentration of selected biomarkers of endothelial dysfunction, the predominant feature of S961-treated mice was a fall in plasma concentration of adiponectin, an adipokine known to exert anti-inflammatory, anti-atherosclerotic properties [48] by multiple mechanisms [39,49]. A fall in plasma adiponectin concentration, as well as fall in adiponectin protein expression in PVAT of TA section, could aggravate locally vascular dysfunction in S961-treated mice [39,48,49]. As regards other biomarkers, only a few displayed significant changes. Of note, the plasma concentration of the biomarkers of glycocalyx disruption, endothelial inflammation, endothelial permeability and hemostasis were also not significantly altered in HFD-fed mice for 2 weeks, despite profound endothelial dysfunction in the AA [14]. In contrast, these biomarkers changed typically in other models of endothelial dysfunction in our previous studies including atherosclerosis and db/db mice [25,30,40]. Accordingly, in the early phase of HFD-induced vascular dysfunction and in early phase of hyperglycemia induced by S961, systemic biomarkers did not indicate severe endothelial dysfunction, emphasizing the insidious nature of the early detrimental effects of PVAT dysfunction on vascular function.

There are some important limitations in the study that need to be discussed. First, to compare our study with previously published data on S961-induced hyperglycemia and effects of HFD, we have used only male mice. Second, limitation of using IRA in an animal model is the systemic effects it elicits, as insulin receptors are abundant in many cell types, including β-cells, adipocytes, hepatocytes, myocytes and endothelial cells. In this sense, this model displays the effect of cumulative action of S961 at different targets. On the other hand, the reversibility of this pharmacological intervention allows for studying the recovery after IRA administration is stopped, which may be of particular interest in the context of cellular memory and epigenetic modification due to hyperglycemia. Nevertheless, these limitations, the profound endothelial dysfunction in S961-treated mice in both the TA and AA related to a direct effect of IRA (insulin receptor blockade and hyperglycemia) as well as indirectly by eliciting altered signaling in PVAT [14] uncovered that altered insulin signaling in diabetes induces not only pronounced endothelial dysfunction as evidenced by numerous previous studies, but simultaneously induces PVAT dysfunction, even in the absence of fat overload and obesity.

## 5. Conclusions

In summary, we demonstrated here that short-term administration of IRA, the S961 peptide, in mice resulted in robust endothelial dysfunction and PVAT dysfunction, characterized here in detailed in the AA and TA showing distinct characteristics as compared with alterations in PVAT in mice fed HFD [14]. In particular, PVAT dysfunction in the TA was associated with abolished thermogenesis in PVAT surrounding the thoracic aorta, decreased adiponectin level while in both AA and TA there was a severely altered NOS/ROS balance. Collectively, PVAT plays a fundamental role in the modulation of vascular function in mouse models of hyperglycemia induced by blockade of insulin signaling, that was not linked to fat load or obesity, known previously to affect PVAT also in humans [50]. Accordingly, PVAT-targeted therapy [51] might offer a novel approach to preserve PVAT-dependent homeostatic and vasoprotective function of perivascular “batokines” to prevent vascular dysfunction in diabetes and insulin resistance.

## Figures and Tables

**Table 1:** Metabolic and biochemical effects of the S961 in mice after 2-weeks of treatment.

Parameter	Vehicle	S961	*p*
Creatinine [µmol/L]	18.8 (16.1–20.0)	11.5 (8.1–16.4) *	0.033
TG [mmol/L]	0.91 (0.74–1.10)	1.34 (0.93–1.64) *	0.029
TC [mmol/L]	2.40 ± 0.18	2.32 ± 0.09	0.67
HDL [mmol/L]	1.309 ± 0.039	1.175 ± 0.026 **	0.0001
LDL [mmol/L]	0.21 (0.19–0.28)	0.15 (0.13–0.23)	0.058
ALT [U/L]	39.89 ± 4.65	57.54 ± 2.82 **	0.003
AST [U/L]	84.18 ± 8.96	94.74 ± 6.64	0.35
LDH [U/L]	754 ± 76	693 ± 65	0.55
HbA1c [%]	5.6 (5.5–5.8)	10.0 (9.1–10.5) ****	<0.0001
Plasma glucose [mmol/L]	12.2 ± 0.8	33.7 ± 1.2 ****	<0.0001
Blood glucose [mmol/L]	6.8 ± 0.6	29.6 ± 0.6 ****	<0.0001
C-peptide 2 [nmol/L]	0.6 (0.4–1.4)	15.5 (14.5–17.8) ****	<0.0001
HOMA-IR_C-peptide_	0.4 (0.2–0.6)	23.1 (19.6–25.7) ****	<0.0001
HOMA-β	1.1 (0.7–5.0)	9.8 (9.3–11.9) ****	<0.0001
GSH:GSSH ratio	30.7 ± 4.5	16.8 ± 2.5 *	0.013

TC—total cholesterol, HDL—high-density lipoprotein, LDL—low-density lipoprotein, TG—triglycerides, ALT—alanine aminotransferase, AST—aspartate aminotransferase, LDH—lactate dehydrogenase, HbA1c—glycated hemoglobin. GSH/GSSG—reduced/oxidized glutathione in red blood cells. HOMA_IR was calculated as (C-peptide 2 × glucose)/22.5 and HOMA-b as 20 × C-peptide-2/(glucose—3.5) [35]. Data shown as mean +/− SEM. Statistical analysis by two-sided Student’s t-test, normality confirmed by Shapiro–Wilk test, variance by F-test, except creatinine, TG, LDL, HbA1c, C-peptide 2, HOMA-IR and HOMA-β which were analyzed using Mann–Whitney U test and are shown as median (25–75 percentile). Vehicle n = 7, S961 n = 10 mice. * *p* < 0.05, ** *p* < 0.01, **** *p* < 0.001.

**Table 2:** Effect of S961 on blood count in mice after 2-weeks treatment.

Blood Count	Vehicle	S961	*p*
White blood cells [10^3^/µL]	2.83 ± 0.18	2.47 ± 0.18	0.19
Red blood cells [10^6^/µL]	9.62 ± 0.24	10.96 ± 0.15 ****	0.000151
Hemoglobin [10^3^/µL]	13.97 ± 0.41	15.96 ± 0.21 ****	0.000248
Hematocrit [%]	50.3 ± 1.50	59.1 ± 0.80 ****	0.000046
Mean corpuscular volume [fL]	52.1 ± 0.50	53.9 ± 0.50 *	0.031
Mean corpuscular hemoglobin [pg]	14.5 ± 0.20	14.6 ± 0.10	0.87
Mean corpuscular hemoglobin concentration [g/dL]	27.8 ± 0.20	27.0 ± 0.20 *	0.028
Platelets [10^3^/µL]	1280 ± 52	1331 ± 58	0.54

Data shown as mean +/− SEM. Statistical analysis by two-sided Student’s t-test, normality confirmed by Shapiro–Wilk test, variance by F-test. Vehicle n = 7, S961 n = 10 mice. * *p* < 0.05, **** *p* < 0.001

## Data Availability

The data presented in this study are available on reasonable request from the corresponding author.
